# Sorghum Flour Features Related to Dry Heat Treatment and Milling

**DOI:** 10.3390/foods12112248

**Published:** 2023-06-02

**Authors:** Ana Batariuc, Ionica Coțovanu, Silvia Mironeasa

**Affiliations:** Faculty of Food Engineering, “Stefan cel Mare” University of Suceava, 13 Universitatii Street, 720229 Suceava, Romania; ana.batariuc@usm.ro (A.B.); ionica.cotovanu@usm.ro (I.C.)

**Keywords:** dry heat treatments, fractionation, functional properties, nutritional properties, sorghum grains

## Abstract

Heat treatment of sorghum kernels has the potential to improve their nutritional properties. The goal of this study was to assess the impact of dry heat treatment at two temperatures (121 and 140 °C) and grain fractionation, on the chemical and functional properties of red sorghum flour with three different particle sizes (small, medium, and large), for process optimization. The results showed that the treatment temperature had a positive effect on the water absorption capacity, as well as the fat, ash, moisture and carbohydrate content, whereas the opposite tendency was obtained for oil absorption capacity, swelling power, emulsion activity and protein and fiber content. Sorghum flour particle size had a positive impact on water absorption capacity, emulsion activity and protein, carbohydrate and fiber content, while oil absorption capacity, swelling power and fat, ash and moisture content were adversely affected. The optimization process showed that at the treatment temperature at 133 °C, an increase in fat, ash, fiber and carbohydrate content was experienced in the optimal fraction dimension of red sorghum grains. Moreover, the antioxidant performance showed that this fraction produced the best reducing capability when water was used as an extraction solvent. Starch digestibility revealed a 22.81% rise in resistant starch, while the thermal properties showed that gelatinization enthalpy was 1.90 times higher compared to the control sample. These findings may be helpful for researchers and the food industry in developing various functional foods or gluten-free bakery products.

## 1. Introduction

Sorghum (*Sorghum bicolor* L. Moench) is one of the most drought-resistant crops, making it the ideal crop for dealing with world food security, and is the fifth most important cereal in terms of production capacity after corn, rice, wheat, and barley [1]. It presents a high resistance to drought conditions and showed the possibility of cultivation in semi-arid and arid lands. In recent years, sorghum production increased significantly to meet the demand of the growing global population for a dependable and low-cost source of energy for animals and humans.

Sorghum kernels are, on average, 4 mm in length, 2 mm in width, and 2.5 mm in thickness, and are generally spherical in shape [2]. The grain contains three different anatomical components in various proportions: 6% pericarp, 10% germ, and 84% endosperm [3]. The pericarp is composed of three distinctive parts: the epicarp, mesocarp, and endocarp. The pericarp of some sorghum varieties contains high levels of tannins, which inhibit protein digestibility [2]. The sorghum endosperm consists of the peripheral, corneal, and floury portions, while the whitefly layer comprises a single layer of rectangular cells with a thick wall, including large amounts of proteins, ash, oil, minerals, and water-soluble vitamins [4]. The outer endosperm beneath the aleurone layer contains globular cells with starch granules integrated into a dense protein matrix of glutelin and prolamine proteins [2]. The germ contains large amounts of oil, protein, enzymes, and minerals [4]. Reports indicated that the high nutritional value of sorghum grains is related to their high starch and protein content, as well as their bioactive compounds [5,6]. As a major component, starch represents 56 to 79% of grain weight, as well as including 70 to 80% amylopectin and 20 to 30% amylose [7,8]. The protein content varies from 6 to 21.1%, with an average of 11%, depending on the agronomic and environmental conditions during grain growth and the genotype [9]. Regarding protein quality, sorghum presents rich essential amino acid content, such as lysine, tryptophan, arginine, methionine, and aspartamide [10]. The germ is a rich source of oil (28%) and protein (16%), with the albumin and globulin proteins accounting for substantial amounts of lysine (32 to 34%) and starch (10%) [7]. Sorghum endosperm has an elevated protein content (80%), of which prolamins account for 67 to 69%, the germ contains 16%, and albumin and globulin account for 32 to 34% [11]. The lysine was also found in the pericarp, and debranding sorghum reduces a substantial amount of the lysine content [11]. Therefore, the use of integral sorghum flour in food products can be an alternative in order to avoid this inconvenience. Sorghum is recognized as a rich source of slowly digestible starch [12], thus having a beneficial effect on digestion and intestinal absorption of carbohydrates. Regarding the nutritional fat value, the linoleic and oleic acids were the major fatty acid constituents of sorghum lipids [13]. Sorghum grains also include a large number of minerals, as well as B vitamins, vitamin E, β-carotene, etc. [14,15]—components which are vital in terms of disease prevention [16]. In addition, sorghum is a rich source of bioactive compounds, containing phenolic acid, 3-deoxyanthocyanidins, proanthocyanins, and carotenoids, as well as a high content of antioxidants [15,17], making it superior to other cereals, such as wheat, barley, millet and rye [18].

Various foodstuffs, such as bread, cookies, biscuits, noodles, cereal bars, and baked goods, can be manufactured using sorghum flour as an alternative to wheat flour as a raw material [15]. At the same time, a number of researchers continue to investigate various processing methods to enhance the sensorial attributes of sorghum products [19]. Processing methods impacted the chemical and techno-functional properties of sorghum grains and sorghum flour [12,20]. Moreover, sorghum composition is influenced by the color of the pericarp and endosperm and the size, hardness and shape of the kernels [21]. Some studies focused on the potential different processing methods that can improve sorghum grains and flour characteristics, as well as on assessing the formulation required to obtain sorghum grain-based food products accepted by consumers, with promising rising health benefits [22,23]. Previous research showed that some treatment methods, such as soaking, heat treatment, freezing, nixtamalization and osmotic treatment, can considerably affect the structure of physical tissues, the content of nutrients and the functionality of sorghum grain [18,24,25], and, therefore, the physico-chemical properties of sorghum flour. Currently, new processing technologies, such as microwave roasting, nixtamalization and irradiation, were applied to enhance sorghum grains’ features [26,27]. Roasting is one method: it uses cooking treatment on grains, and is widely used to enhance their taste and nutritional value for human consumption. This method reduces nutrition loss and decreases the antinutritional factor when applied to optimal conditions [28]. The cooling of the grains after cooking considerably reduces the protein digestibility, which further determines the formation of the resistant starches that form complexes with kafirin proteins, making grains less susceptible to enzyme attack [27]. The roasting process is carried out by applying microwave or dry heat treatment to impact the total levels of phenols and flavonoids, as well as the antioxidant properties of sorghum flour [27,29]. After roasting, the grains were subjected to grinding and sifting to obtain flour with different particle sizes that incorporated different anatomical parts of the grain, indicating varied nutritional profiles [30,31]. The extraction of pigmented matter deposited in the cell walls of the embedding that contains a high concentration of phenolic depends on the size of the sorghum particles and the method used [29]. Particle dimensions also influence flour functionality and finite product quality.

The knowledge of how some physical treatments applied to sorghum grains affect the physico-chemical composition of different flour particle dimensions and their functionality is of importance in manipulating the quality of baked goods. Nevertheless, there is a lack of information about the effect of dry heat treatment and grinding process on red sorghum grains and their influence on flour particles’ dimensions. The heat generated during the dry heat treatment and the frictional heat and mechanical energy needed for grinding can affect the starch’s structural and molecular properties, as well as other sorghum grain components. Particles’ sizes and surface areas can explain some effects, such as water binding, swelling, absorption and solubility. There is scant information on the features of different red sorghum grains’ flour particle sizes when subjected to physical treatments. In addition, the knowledge of the functional properties of sorghum grains flour plays an essential role in explaining the complex interactions between the fractions of components, together with the nature of the environment in which they are associated and investigated. Therefore, the present study aimed to evaluate the effect of dry heat treatment and grinding process on the nutritional profile and functional properties of different sorghum flour particle sizes, as well as to establish the sorghum flour particle size that provides the best nutritional characteristics and functionality when sorghum grains are subjected to optimal dry heat temperature. Additionally, the optimal product was evaluated based on the color, thermal, molecular characteristics, starch digestibility, and bioactive compounds demonstrated via testing.

## 2. Materials and Methods

### 2.1. Materials

Red sorghum kernels (ES Alize hybrid) were purchased from a retailer (Fălticeni, Romania). Firstly, a dry heat treatment was applied to sorghum grains at two temperatures—121 and 140 °C—for 15 min, and the grains were placed on a tray in a uniform layer of 10 mm in a convection oven (Binder ED53 L, Tuttlingen, Germany). Next, a KitchenAid grain mill (model 1065 KGM, Whirlpool Corporation, Benton Harbor, MI, USA) and a vibratory sieve shaker (Retsch AS, Haan, Germany) were used to ground and sieve the grains, respectively. Three types of particle sizes—large (L > 300 µm), medium (200 µm < M < 250 µm) and small (S < 200 µm)—were taken into the study to find the treatment temperature and fraction that give the best composition and functionality. The characterization of the optimal fraction and comparison with the untreated fraction (control) were performed.

### 2.2. Methods

#### 2.2.1. Compositional Analyses

The moisture, protein, fat and ash were determined following the ICC methods. The total dietary fiber was analyzed with a Megazyme kit (K-TDFR-200a 04/17) following the AACC 32-05.01 method. The carbohydrate was calculated based on difference (Equation (1)):

(1) $$\mathrm{Carbohydrates}\left(\%\right)=100\times (\mathrm{protein}+\mathrm{fat}+\mathrm{ash}+\mathrm{fiber}+\mathrm{moisture})$$

#### 2.2.2. Functional Properties

The Water Absorption Capacity (WAC) was established in accordance with the procedure indicated by Cotovanu et al. [32] with amendments. The results were calculated using Equation (2):

(2) $$\mathrm{Water}\mathrm{absorption}\mathrm{capacity}\left(\%\right)=\frac{m_{1}}{m_{0}}\times 100$$

where m_0_ is the flour weight, and m_1_ is the gel weight.

Oil Absorption Capacity (OAC) was established in accordance with the procedure mentioned by Elkhalifa and Bernhardt [33]. OAC was calculated using Equation (3):

(3) $$\mathrm{Oil}\mathrm{absorption}\mathrm{capacity}\left(\%\right)=\frac{m_{1}}{m_{0}}\times 100$$

where m_0_ is the flour weight, and m_1_ is the gel weight.

Swelling Power (SP) was determined in accordance with the method described by Elkhalifaand Bernhardt [33] with modifications. The supernatant was picked up with caution, the flour-filled sediment was weighed and SP was calculated using Equation (4):

(4) $$\mathrm{SP}\;(g/g)=\frac{\mathrm{swelled}\mathrm{gel}}{\mathrm{sample}\mathrm{weight}}$$

Emulsion Activity (EA), which was determined in accordance with the procedure described by Elkhalifa and Bernhardt [33], was calculated using Equation (5):

(5) $$\mathrm{Emulsifying}\mathrm{activity}\;(\%)=\frac{\mathrm{height}\mathrm{of}\mathrm{emulsion}\mathrm{layer}}{\mathrm{height}\mathrm{of}\mathrm{whole}\mathrm{layer}}\times 100$$

#### 2.2.3. Optimization Process

To evaluate the effect of dry heat temperature and fractions dimensions on the physico-chemical and functional characteristics of sorghum particle sizes, the response surface methodology (RSM) was used. The RSM technique is adequate for analyzing the relationships between several independent factors from the process and their impact on the responses of interest for the studied process using a small number of experiments. This outcome was achieved by obtaining an adequate response surface model for each evaluated response and imposing some conditions that maintain these response models in the desired range. D-optimal design (Design Expert, Stat-Ease, Minneapolis, MN, USA, trial version) was used to estimate the effect of two factors—the process temperature (121 and 140 °C) and the fraction dimension (L > 300, M 200–250, and S < 200 µm)— on some responses, such as protein, fat, ash, moisture, carbohydrates and fiber of sorghum flour, as well as water absorption capacity (WAC), oil absorption capacity (OAC), swelling power (SP), emulsion activity (EA) and functional properties. The value ranges of the responses from the data matrix are shown in Table 1.

The regression analysis was conducted, and the factors’ influence and interaction with the interest responses were assessed via variance analysis (ANOVA), with a 95% confidence level set for the mathematically adapted model for each variable. The adequacy of the predictive model was evaluated through the *F* sequential test, lack of fit (*LoF*), adjusted coefficients of determination (*Adj.-R*^2^), predicted coefficients of determination (*Pred.-R*^2^), and adequate precision (*Adeq. Precision*), which measured the signal-to-noise ratio. The desirability function was applied to find the optimal treatment temperature for the sorghum flour particle size that presents the best nutritionally profile and functionality. In this sense, some constraints, consisting of the maximization of fat, ash, fiber and oil absorption capacity, were applied, with the rest of the variables being maintained within the range. For the predictive model validation, the optimal values predicted were verified. The results were compared to those corresponding to the control sorghum flour particle size. Further characterization of the optimal sample compared to the control was performed.

#### 2.2.4. Characterization of Optimal Sample

*Color Parameters*

Color measurement of sorghum fractions was performed through reflectance with a chromameter CR-400 (Konica Minolta, Inc., Tokyo, Japan). After the calibration with a white reference tile, the parameters measured according to the CIELab system, in triplicate, were lightness (L*), redness/greenness (+a*/−a*) and yellowness/blueness (+b*/−b*). Additionally, Chroma (C*), the total color difference (ΔE) and brown index (BI) were measured [27,34].

*Thermal analysis*

A differential scanning calorimeter DSC 25 (TA Instruments, New Castle, DE, USA) was used to determine the thermal properties in triplicate. The sample used for analysis was obtained by mixing flour with water at a 1:2 (*wt*/*wt*) ratio and maintaining it for 2 h at 25 °C prior to analysis. A small amount of sample (4–5 mg) was weighted into an aluminum mold, covered with a lid, sealed and perforated and placed in the instrument, along a blank tray for reference. The samples were equilibrated for 5 min at 25 °C, after which the temperature was increased to 10 °C (initial temperature), followed by an increase to 140 °C at a heating rate of 5 °C/min using nitrogen as the purge gas at a flow rate of 20 mL/min. The initial temperature (T_i_), gelatinization temperature (T_g_) and final temperature (T_f_) were determined. Additionally, the gelatinization enthalpy was recorded using the thermal system software, and the gelatinization temperature range (T_r_), which was like a difference between the final and initial temperature (T_f_ − T_i_) of gelatinization, was calculated.

*Starch Fractions*

The starch fractions were analyzed in accordance with the international AOAC 2017.16 protocol with a Megazyme kit (K-DSTRS; Megazyme, Bray, Ireland). Using spectrophotometric methods, the rapidly digestible starch (RDS), slowly digestible starch (SDS), resistant starch (RS), total digestible starch (TDS) and total starch contents of sorghum flour fractions were determined in triplicate. Moreover, the starch digestion rate index (SDRI), which is an indicator of in vitro starch digestibility, was calculated as RDS divided by TS. For each sample, two independent preparations were carried out.

*Total Polyphenols and Antiradical Activity*

To prepare the extract, 1 g flour was mixed with different reagents—methanol 99.9%, ethanol 98%, and water, respectively—in a ratio of 1:20 *w*/*v*, before being sonicated for 10, 15, 20 and 30 min at 50 °C and 40 kHz. This extract was used for the determination of total phenolic and antioxidant activity.

For the Total Polyphenol Content (TPC) analysis, 0.2 mL of the extract was added to 2 mL of Folin–Ciocâlteu reagent and 1.8 mL of sodium carbonate (7.5%), and the sample was rested and incubated for 30 min at room temperature in the dark. After this time, the absorbance was measured at 750 nm.

The antioxidant capacity of sorghum fractions was evaluated with 2,2 diphenyl-1-picrylhydrazyl (DPPH). In total, 2 mL of DPPH reagent was mixed with 2 mL of the prepared extract at different time intervals, and the absorbance was read at 517 nm after 30 min of incubation at room temperature. The DPPH AA was calculated using Equation (5):

(6) $$\mathrm{DPPH}\mathrm{AA}\left(\%\right)=(1-\frac{A_{sample}}{A_{blank}})\times 100$$

*Fourier Transforms Infrared (FTIR) Spectrometric Analysis*

The ATR FT-IR spectra of the optimal and control sorghum flour fractions were registered in triplicate as being between 650 cm^−1^ and 4000 cm^−1^, with a resolution of 4 cm^−1^ and 32 scans (Thermo Scientific Nicolet iS20, Waltham, MA, USA). The molecular characteristics were determined by reporting previous peak areas stated in the literature [35,36] and using OMNIC software (9.9.549 version, Termo Fisher Scientifc, Waltham, MA, USA).

### 2.3. Statistics

Student’s *t*-test was applied to test the differences among the experimental and predicted values for the optimal sample at a 95% confidence level. ANOVA and Tukey’s test were used to check the differences between the optimal and control samples. The XLSTAT for Excel 2022 version (Addinsoft, New York, NY, USA) software was used for the statistical tests and Pearson’s correlations matrix.

## 3. Results and Discussions

### 3.1. Proximate Composition and Functional Properties

#### 3.1.1. Effect of Factors

**Figure 1:**
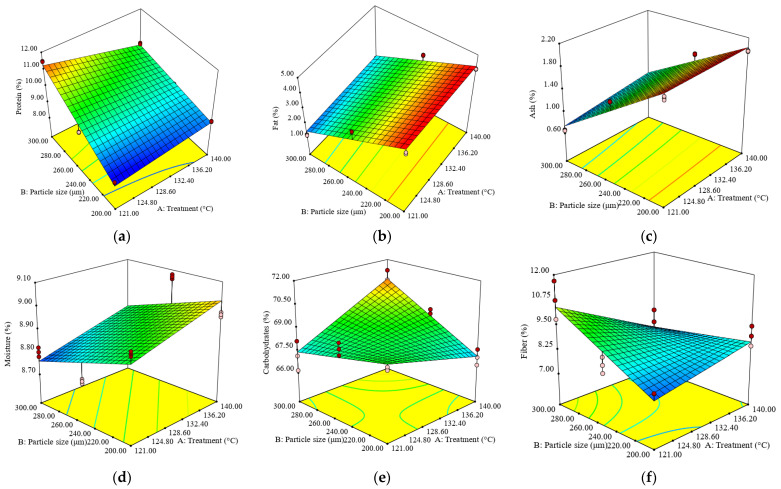
Response surface graph for combined effects of sorghum flour particles’ sizes and treatment temperatures on following aspects: (**a**) protein, (**b**) fat, (**c**) ash, (**d**) moisture, (**e**) carbohydrates and (**f**) fiber.

**Figure 2:**
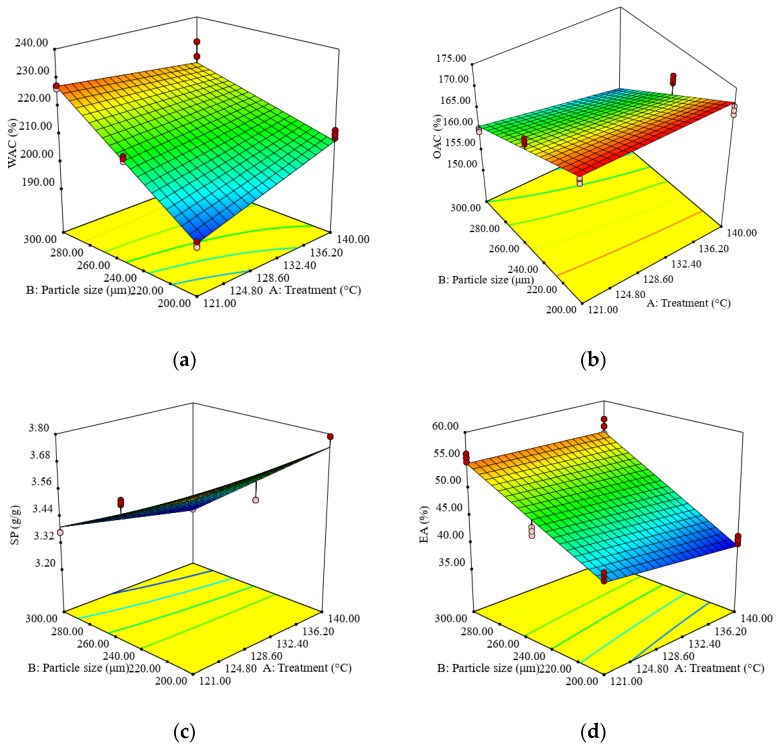
Response surface graph for combined effects of sorghum flour particle size and temperature treatment on following aspects: (**a**) water absorption capacity (WAC), (**b**) oil absorption capacity (OAC), (**c**) swelling power (SP) and (**d**) emulsion activity (EA).

According to the D-optimal design, a total of 18 experimental runs were performed, and the results of targeted responses i.e., protein, fat, ash, moisture, carbohydrates, fiber content, WAC, OAC, SP and EA, are shown in Table S1. Every experimental run was performed in triplicates, and the average results are shown in the Supplementary Materials. Predictive models equations with omitted insignificant regression coefficients for the physico-chemical and functional properties of sorghum fractions are shown in Table 2. The ANOVA reveals that the regression models obtained for the physico-chemical composition and functional properties of sorghum flour are statistically significant at a 95% confidence level, and the satisfactory determination coefficients (*R*^2^ > 0.56) were obtained. Moreover, acceptable adjusted determination coefficients (*Adj.-R*^2^ > 0.60) provided appropriate representation of the experimental data and demonstrated that these models could be used to explain more than 60% of the variability in the responses; only the model for moisture content (*Adj.-R*^2^ = 0.50) explains only 50% of the data variability. The lack of fit testing only confirmed the adequacy of the model for carbohydrates, since the *LoF* coefficient for this response had an insignificant *p*-value (*p* > 0.05). Ideally, the *F*-ratio for lack of fit needs to be non-significant. Unfortunately, this outcome does not guarantee that the model will be satisfactory as a prediction equation [37]. *Adj.-R*^2^ and *Pred.-R*^2^ have a reasonable level of agreement (within 0.2) with each other. All of the predictive models can be used to navigate the design space due to the ratio value being greater than four, which is desirable, indicating an adequate signal for all of the models. Based on this information, as well as the reasonable values obtained for several statistical indicators reported (*Adj.-R*^2^, *Pred.-R*^2^, and *Adeq. Precision*), we considered that the models are satisfactory in ensuring the efficiency of the optimization. The response area graphs showed the variability in the studied parameters with treatment temperatures and fraction dimensions for sorghum flour, thus helping to visualize the shapes of the contours and providing useful evidence on the model’s suitability (Figure 1 and Figure 2).

The treatment temperature (A) and the interaction between treatment temperature and particle size (A × B) have a negative significant effect (*p* < 0.05) on the protein content of sorghum flour, while the particle size (B) had a positive impact, being the most linear effect (Table 2). An increase in particle size (B) raised the protein content of sorghum flour. The fat, ash and moisture content followed an opposite trend as particle size (B) increased. The treatment temperature (A) had a strong influence (*p* < 0.05) on ash and moisture content (Table 2). Both factors (A, B) and the interaction between them (A × B) significantly (*p* < 0.05) influenced the carbohydrate content of sorghum flour in a positive way, with the interaction between factors showing the highest effect on carbohydrates. An increase in sorghum flour carbohydrates was observed when particle size and temperature increased (Figure 1). For the fiber content of sorghum flour, particle size (B) had an important positive effect, while factor A and the interaction between factors had a negative effect, where the interaction effect was higher on fiber content.

The response surface plots of ash and carbohydrates with variation in treatment temperature and particle size of sorghum showed that sorghum flour exhibited a significant (*p* < 0.05) decrease in protein and fiber, as well as an increase in carbohydrates with the treatment temperature rise, while fat, ash and moisture decreased with the rise in particle size (Figure 1). On the other hand, the increase in particle size determined a significant (*p* < 0.05) rise in protein, carbohydrates and fiber. The interaction between factors (A × B) had a significant effect on the protein, carbohydrate and fiber content. It also determined a decrease in the protein and fiber content of sorghum flour, as well as a rise in carbohydrate content.

Water absorption capacity was significantly (*p* < 0.05) affected by treatment temperature (A) and particle size (B), with the last factor having the highest effect, while their interaction (A × B) negatively affected WAC (Table 2). A rise in WAC of sorghum fractions may be caused by the development of a porous structure and damaging starch, such as gelatinization, when roasting grains, reflecting their water binding and the loss of organized starch polymers [38].

The oil absorption capacity (OAC) of sorghum flour significantly (*p* < 0.05) decreased with the increase in treatment temperature and particle sizes, as can be seen in Figure 2. The decrease in OAC of sorghum flour, likely due to the application of microwave treatment, was reported by Almaiman et al. [39]. This decrease in oil absorption capacity probably occurs due to changes in proteins, the hydrophobic characteristics of macromolecules and the amino acid quantities [40]. Obasi et al. [41] also reported that a reduction in polar amino acids, a change in their polarity or denaturation and dissociation of the constituent protein can explain the lower OAC. The results obtained are supported by some studies on sorghum and flaxseed that showed a decrease in OAC upon roasting [27].

The swelling power (SP) parameter of sorghum flour was significantly (*p* < 0.05) negatively affected by the particle size (B), as well as the interaction between factors (A × B); however, the highest influence was observed for the particle size. As a consequence of milling, the components of sorghum kernels change, thereby altering their ability to bind water and release soluble components [42,43].

The emulsion activity (EA) was significantly (*p* < 0.05) affected by the particle size (B), with the increase in particle size determining a rise in EA. Protein dispersion at the surface tension in water and air through its structural deployment affected the EA [44].

#### 3.1.2. Pearson’s Correlations between Variables

Significant correlations were noticed between some of the studied characteristics of sorghum flour (Table 3). With respect to correlations between functional properties and chemical characteristics, water absorption capacity had a positive correlation with protein (r = 0.841, *p* < 0.01) and fiber content (r = 0.537, *p* < 0.05), as well as a negative correlation with fat (r = −0.843, *p* < 0.01) and ash (r = −0.843, *p* < 0.01). The various quantities and types of lipids, proteins and carbohydrates can affect the WAC due to their separate polarity and, therefore, different water binding and absorption capacities [45]. OAC was positively correlated (r > 0.85, *p* < 0.01) with fat and ash, as well as negatively correlated with protein content (r = −0.692, *p* < 0.01). Oil absorption capacity is defined by the physical trapping of oil and the binding of fats to the molecules of apolar proteins, and is dependent on lipophilia, amino acid composition and surface polarity [12]. The WAC and OAC of the dough are important because these two properties greatly influence the moisture, texture and appearance of the product. Significant correlations were found between SP and protein content (r = −0.694, *p* < 0.01), as well as carbohydrates (r = −0.576, *p* < 0.05), which confirms that the carbohydrates–protein compounds have a higher emulsifying capacity, with their ability to stabilise the interfaces being provided to the isoelectric point of the proteins [46]. EA was indirectly negatively correlated with fat (r = −0.971, *p* < 0.01), ash (r = −0.972, *p* < 0.01) and moisture (r = −0.600, *p* < 0.01), as well as positively correlated with protein (r = 0.852, *p* < 0.01) and fiber (r = 0.570, *p* < 0.05). An increased emulsion capacity can be achieved by increasing the balanced surface capacity of the hydrophobic and hydrophilic protein sites [45].

#### 3.1.3. Optimization and Model Validation

Following the optimization process for each sorghum flour fraction, it was found that the dry treatment at 133 °C was appropriate for the M fraction (Table 4) to achieve the desired functional and nutritional properties.

The optimal sample revealed significantly (*p* ˂ 0.05) higher values for fat, ash, fiber, WAC, OAC and SP than Control M. Only moisture content presented a lower value for the optimal sample compared to the control. These results may be attributed to the biochemistry and morphology of sorghum grains, as well as to the milling process that induces modification in the components, probably because of photo-oxidation and limited bioavailability of compounds [47]. The results are supported by previous studies, which stated that sorghum grains subjected to thermal treatment, soaking and steaming undergo important changes in their physical tissue structure, nutrient levels and functional characteristics [48].

### 3.2. Characterization of the Optimal Sample

#### 3.2.1. Color Parameters

The results for color parameters showed that the L*, a* and b* of optimal and control samples differ significantly (Table 5). The optimal fraction exhibited lower color tone values compared to the control. The reduction in lightness can be associated with reduced moisture content, as can be seen in Table 3, and the development of a glazed surface, in line with the evidence that Sharanagat et al. [27] affirmed. Similarly, a decrease in a* and b* values was noticed in the optimal sample compared to the control, showing that dry heat treatment determined a decrease in the degree of redness and yellowness nuances. Chroma and brown indeces (BI) exhibited strong differences (*p* < 0.05) in the optimal and control fractions. The total color difference value (1.72) indicates that there are distinct differences in terms of perceivable color (1.5 < ΔE < 3) [49].

The variations in color parameters on optimal M samples may be ascribed to biochemical processes, polyphenol leaching during dry heat treatment and fractionation. Similar findings were reported by Taylor and Duodu [50] when they studied the effect of sorghum and millet processing on phenolic phytochemicals. The amount and type of phenols and metal ions present in the kernels had an impact on the color of sorghum products [51].

#### 3.2.2. Functional Groups

**Figure 3:**
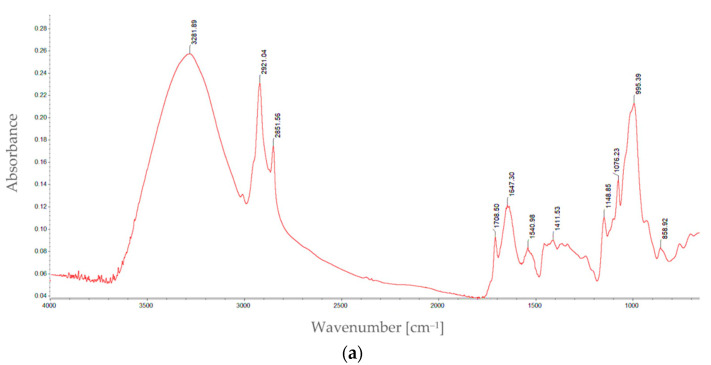

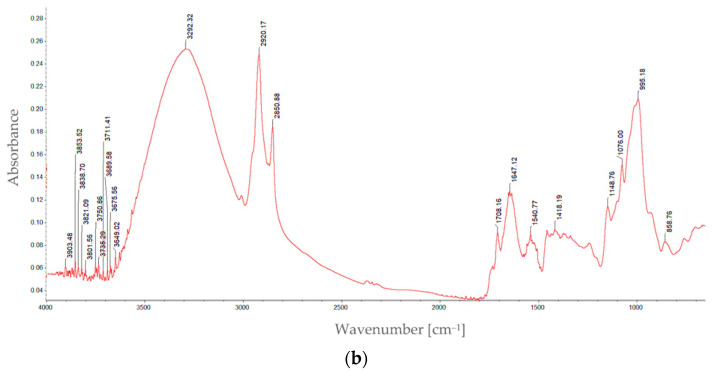
FT-IR spectra of control (**a**) and optimal (**b**) sorghum particle sizes.

The findings of the FTIR analysis of the control and optimal sorghum fractions are presented in Figure 3a,b. Specific spectral regions in the control and optimal samples showed variations in the absorbances and signal heights of the sample spectra regarding the different types of binding stretching, which were interpreted based on the literature data [21].

FTIR was applied to highlight the structural changes in the proteins during dry heat treatment. A significant influence of dry heat treatment was indicated in the regions 3500–4000 cm^−1^. The intensity of peaks, especially the two peaks at 3711.41 cm^−1^ and 3953.52 cm^−1^, demonstrates the distribution of functional groups because of the dehydroxylation reaction and the increase in the WAC that occurs during dry heat treatment. As a result of the dehydroxylation reaction, the water produced favors changes in the water activity of food and the mobility of various reactants [27]. Moreover, the region that appeared at 3600 cm^−1^ and 3800 cm^−1^ indicates the O-H groups of alcohol/phenols intermolecularly bonded, and the changes in peak intensities indicate the degree of formation of hydrogen bonds [52]. In the spectral range 3000–3500 cm^−1^ that indicates changes in the N-H activity of primary and secondary amines, as well as the O-H of carboxylic acid, alcohols (intra-molecular bonded) or starch, the prominent absorption peaks are not higher in the optimal sample than in the control (Figure 3). The intensities of two peaks (2850.88 cm^−1^ and 2920.17) at the region 2800–3000 cm^−1^ for the optimal sample changed compared to the corresponding control. This result was due to the stretching of the C-H cis-olefinic or C-H aldehyde bonds and changes in alkenes, lipid and olefinic composition upon dry heat treatment, as stated by Sharanaga et al. [27]. Changes in the –NH^3+^ amines or hydrohallides and –PH in the phosphine functional groups can be observed in the 2400 to 2300 cm^−1^ region. The region 1200 to 1900 cm^−1^ indicates various functional groups and compounds, such as amides, amino acids, –C=O in aldehydes, C–O in esters, CˆO in anhydrides, =O in lactones, t-butyl groups, N–O pyridine groups, esters, lactones, etc. [27]. In this interval, some peaks have higher intensities in the optimal sample than in the control. Similar changes in these regions were also reported for proso-millet [53]. The peak intensities showed different variations in the region 600–1200 cm^−1^. The prominent peaks were sighted at 1418.19 cm^−1^ and 1647.12 cm^−1^ in the optimal sample and at 1411.53 cm^−1^ and 1647.30 cm^−1^ in the control sample, while peak intensity decreased from 1540.98 to 1540.77 cm^−1^, indicating the loss of compounds in the optimal sample. Changes in region 900–1100 cm^−1^ were also reported by Sun et al. [53], which occurred when proso millet underwent a dry heat treatment. These changes were caused by the angular deformation of the C–H bond in the flour, the skeletal vibration of −1–4 glycosidic bonds (C–O–C), the formation of new groups, and the stretching vibration of the C–O bond in the esters produced between the non-starch constituents, such as –COOH, in the protein and starch molecules (hydroxyl groups), further supporting the results of this study [52]. The optimal fraction differed from the control fraction at 3292, 2920/2850 and 600–1700 cm^−1^, primarily due to the changes in both the functional groups of protein, starch and phenolics and their bonding. The band’s heights at 858 cm^−1^ can characterize the substitutions in aromatic rings (aromatic C–H bonds). The information-rich fingerprint region from 900 to 1500 cm^−1^ can characterize amylose–lipid complexes present in the whole grain, amide III (1230–1330 cm^−1^), or structural carbohydrates, such as starch and cellulose (unsaturated bonds C=C connected to the oxygen atoms O–C=C or the nitrogen atoms N–C=C) [27]. The observed band’s heights at 1646 cm^−1^ and 1540 cm^−1^ are correlated with proteins [54]. The samples exhibited a strong band at 1646 cm^−1^, which was consistent with the presence of the α-helix structure of the protein. The characteristic band at 1540 cm^−1^ for the β-sheet structure was buried under the strong α-helix band centered at 1646 cm^−1^. A β-sheet structure may develop during heat treatment and may be a factor in making sorghum protein less digestible and harder to extract. The spectral data of group O─H made it possible to identify phenolic compounds, such as flavonoids and tannins, and is sensitive to subtle structural variations [55,56].

#### 3.2.3. Thermal Characteristics

DSC analysis showed that control and optimal sorghum fractions exhibited a single endothermic transition, with corresponding temperatures and enthalpies shown in Figure S1. A significant difference between the samples, in terms of initial (Ti), gelatinization and final (Tf) temperatures, as well as in the ΔT_r_, and ΔH values, was observed (Table 6).

The initial, gelatinization and final temperatures (Ti, Tg, Tf), as well as ΔH, were affected by the interactions between the starch and sorghum proteins during the heating stage. For the optimal fraction, an increase in T_i_, T_f_ and ΔH could be observed compared with the control fraction. The results indicate that optimal sorghum fraction flour requires high temperatures to initiate gelatinization at 69.82 °C compared to the control fraction (69.03 °C). The T_g_ values of control and optimal samples exhibited close peak melting, showing the precise point where starch granules present in the samples broke into smaller units. The gelatinization temperature was closer to the results reported by Ahmed et al. [57]. Related to the final temperature, the results showed that the optimal fraction exhibited a higher value (82.07 °C) than the control fraction (79.58 °C). The values were closer to those reported by Ahmed et al. (2016), but lower than the results reported by Olamiti et al. [58] for the malting and fermentation of sorghum flour. According to the obtained results, it could be depicted that the optimal sorghum fraction had a higher gelatinization range (12.25 °C) than the control fraction (10.55 °C). The rise in the gelatinization range could be due to protein content, as well as the crystal starch structure as stated by Yang et al. [59]. An increase in a starch–lipid complex formation diminishes the degree of hydration in the amorphous area, thereby determining the amount of thermal energy necessary for melting [58,60]. Gelatinization ranges (ΔTr) values are correlated to the results of malting and fermentation of sorghum flour reported by Olamiti et al. [58], as well as those reported by Jideani and Scott [61] for cooked pearl millet flour. The optimal sorghum fraction recorded relatively high enthalpy compared to the control fraction, exhibiting the highest energy used to melt starch granules. The enthalpy results of the optimal and control sorghum fraction were lower than the results reported by Ahmed et al. [57] for sorghum starch. Variations in the final temperature and enthalpy in the optimal sorghum fraction were attributed to starch gelatinization, which might be hindered by the presence of protein, melting enthalpy, starch structure and lipid–starch complexes [62,63]. Gelatinization of sorghum flour fraction brings disruption or a collapse in molecular granules with irresistible changes in properties such as granular swelling, native crystallite melting, loss of birefringence and starch solubilization [64]. The variations in thermal properties could be attributed to starch–protein complexes that form unbroken granules that are difficult to break during processing, thus requiring high energy to gelatinize [58,65].

#### 3.2.4. Starch Digestibility

Starch digestibility regarding rapidly digestible starch (RDS), slowly digested starch (SDS), total digestible starch (TDS), resistant starch (RS), total starch (TS) and the starch digestibility rate index (SDRI) presented significant differences between optimal and control samples of sorghum flour (Table 7). For the optimal sample, the values of RDS, SDS, TDS, RS and TS were higher.

The starch fraction content of cereal-based food products is especially studied by researchers due to the implications of these fractions for the control of blood glucose and insulin levels in humans. Studies on starch digestibility showed that sorghum possesses low starch digestibility as determined by the types and content of phenolic compounds. As can be seen from Table 6, the SDS and RS increase significantly (*p* < 0.05) in the optimal sample compared to the control. This increase can be related to the polyphenols from the optimal flour, which are able to decrease the starch digestibility by means of inhibition of enzymes and/or interactions with starch molecules, in accordance with findings from previous works [66,67]. Additionally, it was reported that the disulphide bond cross-linked matrix of kafirins may interact with sorghum starch granules, contributing to the decrease in starch accessibility to enzymes and/or acting as a barrier to starch gelatinization [66,68]. Moreover, a synergistic inhibitory effect against starch-degrading enzymes determined by the summed effect of the different polyphenols and kafirins from sorghum was reported [66]. However, the optimal fraction possessed a higher TS content than the control. The most useful tool in predicting the glycemic index of foods based sorghum with different TS content is SDRI. The optimal fraction possessed a low SRDI, while RS increased by above 22% compared with the control, which represents a confirmation that foods based on this sorghum fraction will have a low glycemic index. Moreover, taking into account the high content of polyphenols from the optimal sample and their ability to act as a potential inhibitor of digestive enzymes, this sorghum flour fraction could be used to formulate novel functional foods.

#### 3.2.5. Antiradical Activity and Total Polyphenols

The results presented showed that the free radical scavenging activity of the optimal sample was higher (77.33–91.34%) than the control sample (46.98–86.88) (Table 8). Regarding the solvent type, water exhibited the highest rate of antioxidant activity for the optimal sample (91.34%), while ethanol obtained the lowest activity (77.33%). The results showed a significant increase in antioxidant activity in the extracts with water with respect to the extraction time.

The results of total phenolic content revealed that the optimal sample contains a higher value of TPC, whereas the control exhibited a lower potential. For the optimal sample, the highest TPC content was obtained when methanol was used (24.85 mg GAE/100), and the lowest content was obtained with water (12.21 mg GAE/100 g). There were significant differences (*p* ˂ 0.05) between all of the solvent types. With the increase in time extraction, an increase in TPC content was observed in the following order: Water ˂ Ethanol ˂ Methanol.

The results indicated that higher levels of phenolics are present in the optimal fraction than in the control fraction, showing that dry heat treatment at a temperature for 15 min of 133 °C determined an increase in the total phenolics content (TPC). The TPC increase is probably due to the destruction of the internal tissue in these dry heat treatment conditions. This increase could be associated with the formation of phenolics other than endogenous examples, due to the dissociation of conjugated phenolic moiety during thermal processing, followed by some polymerization and/or oxidation reaction. Additionally, the Maillard reaction and chemical oxidation of phenols could also contribute to the rise in total phenolics content [69]. Moreover, the extraction solvent type and the extraction time become key factors in the quantification of total phenolic content (TPC). Among all of the solvents, methanol gives the best yield, followed by ethanol and water, confirming that the extraction solvent has significant impacts on the release efficiency of phenolics. It was found that the amounts of phenolics released via the water extraction method were very low, ranging from 11.79 mg GAE/100 g d.m. at 10 min extraction time to 13.84 mg GAE/100 g d.m. at 30 min extraction time. The methanol extraction significantly improved the release of phenolics in both optimal and control fractions (*p* < 0.05), and the total contents reached up to 17.59 mg GAE/100 g d.m. for the control fraction and 24.85 mg GAE/100 g d.m. for the optimal fraction at 30 min extraction time. The polyphenolic contents of the ethanolic extracts in the optimal and control fractions were relatively higher compared to those of the extracts in water. These results could be associated with the solvent polarity and the solubility of polyphenolic compounds in sorghum [70]. Furthermore, Zhang et al. [71] used ethanol combined with water, i.e., 50–100% (*v*/*v*), to extract more phenolic compounds from plants. In another study, it was reported that the sorghum extract using 50% ethanol and 50% water exhibited the highest total polyphenol content among the sorghum extracts using different ethanol concentrations (50%, 80%, and 100%) [72]. The TPC of sorghum extract increased with decreasing ethanol concentration. Han et al. [73] reported that TPC in 60% ethanol extract in sorghum grain was higher than in other ethanolic extractions. In another study, a 53% ethanol concentration was confirmed as the optimized ethanol concentration via response surface methodology in obtaining the high total polyphenol content in sorghum extract with 30–70% ethanol concentration [74].

On the other hand, regardless of the type of solvent, a rise in the total phenolic content with an extraction time increase was observed. The highest level (24.85 mg GAE/100 g d.m.) of polyphenolic contents was found at 30 min extraction time in methanolic extract. For all of the studied samples, the highest yield in total phenolics content was obtained at 30 min extraction time, confirming that this time is more effective than 10 or 20 min for releasing phenolics compounds. The results obtained were in line with those sourced from previously published works [75,76] when an increase in total phenols in heat-treated red sorghum compared to raw sorghum was reported. An increase in the soluble total phenols content in sorghum after roasting for 5 min at 150 °C compared to the control sorghum was also reported by Hithamani and Srinivasan [77]. In contrast, another research group [48] reported losses of total phenolic content when sorghum was subjected to 150 °C heating for 60 min.

In the present research, the antioxidant capacity of the phenolic extracts at 10, 15, 20 and 30 min in the optimal and control sorghum fractions, following different solvent extraction methods, were evaluated based on the radical scavenging activity of DPPH. It was reported that polyphenols possess antioxidant activity due to the ability of its phenolic ring to stabilize free radicals [78]. As previous studies stated, sorghum phenolic compounds are linked to high antioxidant capacity and their related health benefits [26,67]. The antioxidant capacity results indicated higher values for the fractions from heat-treated red sorghum grains compared to the control sorghum, except when using methanol as an extraction solvent (Table 7). In general, appropriate heat treatment of raw materials increases the antioxidant composition [79]. The increase in antioxidant activity after dry heat treatment is associated with the production of the antioxidant melanoidin compounds through browning reactions, such as the Maillard reaction [80].

As natural phenol components exhibit radical scavenging activity [81], the increase in the radical scavenging activity of the optimal sample is probably due to a dry heat treatment-induced increase in phenolic compounds. Even though the methanolic extract presented the highest phenolics content, the scavenger radical DPPH activity was lower compared to the scavenger radical DPPH activity of the extract with water. The extracts in water showed relatively high radical scavenging activity compared to the methanolic and ethanolic extracts (Table 7). This result is likely due to the amounts of pigments from red sorghum extracted in water that possess strong antioxidant activity. The results are consistent with those of Choi et al. [81], who reported relatively high radical scavenging activity in red sorghum (92%) and black rice (87%) compared to non-pigmented grains.

As is shown in Table 8, the DPPH value was evidently higher when water was used in extraction, varying with time from 91.34% to 94.65% d.m., than when methanol or ethanol was used. These greater values for antioxidant activity can be related to some phenolic compounds being present in an extract with water that possesses strong antioxidant activity. Some researchers stated that ellagic acid and quercetin possessed the stronger antioxidant activity revealed via the in vitro/vivo antioxidant assay [82]. Stefoska-Needham et al. [83] affirmed that high molecular weight oligomers or polymers of condensed tannins in sorghum exhibit strong radical-scavenging activity, inhibiting the pro-oxidative enzymes. Methanol extraction can promote the release of bound phenolic compounds in sorghum fractions; however, it probably also resulted in the loss of some phenolic compounds with higher antioxidant activity, such as gallic acid, ellagic acid and quercetin.

## 4. Conclusions

Dry heat treatment of sorghum kernels and fractionation remarkably influenced the nutritional and functional characteristics of sorghum flour. Treatment temperature increase determined the increase in carbohydrates, ash content and water absorption capacity, while protein content, fiber and oil absorption capacity decreased. A rise in fat content, ash, moisture, oil absorption capacity and swelling power was noticed when the fraction dimension was reduced. The dry heat treatment of sorghum grains at 133 °C indicated the optimal characteristics for medium fraction dimension in terms of nutritional and functional properties. The optimal sample showed lower lightness and red and yellow nuances compared to the control samples. Thermal properties suggested that dry heat treatment induced changes in protein and starch structure, meaning that the optimal sample required higher temperatures to initiate gelatinization and exhibited a higher gelatinization range than the control sample. Starch digestibility analysis revealed that the optimal sample presents a rise in slowly digested starch and resistant starch, while the starch digestion rate index decreases. The total phenolic content and antiradical activity of the optimal treated sample were higher than in the control sample. Hence, the optimal treatment temperature of sorghum grains and optimal fraction dimension obtained in this study could be used as a standard for the improvement of sorghum flours for food processing companies. Moreover, these results will help researchers and producers to further the development of value-added gluten-free products with the best quality parameters.

## Figures and Tables

**Table 1:** Minimum and maximum values of responses from experimental design.

Variable	Minimum Value	Maximum Value
Protein (%)	8.48	11.51
Fat (%)	1.12	4.16
Ash (%)	0.65	1.98
Moisture (%)	8.74	9.08
Carbohydrates (%)	66.16	71.25
Fiber (%)	7.38	11.70
Functional properties		
WAC (%)	191.11	230.47
OAC (%)	152.99	171.60
SP (g/g)	3.32	3.79
EA (%)	39.78	56.41

WAC—water absorption capacity, OAC—oil absorption capacity, SP—swelling power, EA—emulsion activity.

**Table 2:** Corresponding coefficients in predictive models for physico-chemical composition and functional properties of sorghum flour.

Factor	Protein (%)	Fat (%)	Ash (%)	Moisture (%)	Carbohydrates (%)	Fiber (%)	WAC (%)	OAC (%)	SP (g/g)	EA (%)
Constant	9.56	2.96	1.44	8.89	68.51	8.64	212.15	165.03	3.53	47.58
A	−0.20 **	ns	0.07 *	ns	0.38 *	−0.41 *	2.82 **	−1.36 *	ns	ns
B	0.99 ***	−1.35 ***	−0.57 ***	−0.07 *	0.52 *	0.47 *	12.60 ***	−6.86 ***	−0.19 ***	6.63 ***
A × B	−0.49 ***	ns	ns	-	1.24 ***	−0.89 ***	−5.26 ***	ns	−0.04 *	ns
Model fitting										
*p_model_*	<0.001	<0.001	<0.001	<0.05	<0.001	<0.001	<0.001	<0.001	<0.001	<0.001
*p* ^*†*^_*LoF*_	*	*	*	*	ns	*	*	*	*	*
*Adj.-R* ^2^	0.92	0.91	0.95	0.50	0.79	0.61	0.91	0.86	0.89	0.90
*Pred.-R* ^2^	0.90	0.88	0.93	0.40	0.69	0.46	0.88	0.83	0.86	0.87
*Adeq. Precision*	22.37	16.81	23.86	7.96	12.29	8.01	20.85	14.69	17.00	17.22

* *p* < 0.05, ** *p* < 0.01, *** *p* < 0.001, ns—insignificant term, *^†^_LoF_*—lack of fit, WAC—water absorption capacity, OAC—oil-absorption capacity, SP—swelling power, EA—emulsion activity, A—treatment temperature factor, B—particle size factor.

**Table 3:** Pearson’s correlation coefficients.

Variables	Protein	Fat	Ash	Moisture	Carbohydrates	Fiber	WAC	OAC	SP	EA
Protein	1									
Fat	−0.918 **	1								
Ash	−0.930 *	0.994 **	1							
Moisture	−0.501 *	0.539 **	0.596 **	1						
Carbohydrates	−0.159	−0.178	−0.158	−0.018	1					
Fiber	0.745 **	−0.502 *	−0.522 *	−0.372	−0.439	1				
WAC	0.841 **	−0.849 **	−0.843 **	−0.426	0.109	0.537 *	1			
OAC	−0.692 **	0.908 **	0.878 **	0.461	−0.432	−0.203	−0.806 **	1		
SP	−0.694 **	0.858 **	0.847 **	0.223	−0.576 *	−0.125	−0.699 **	0.839 **	1	
EA	0.852 **	−0.971 **	−0.972 **	−0.600 **	0.328	0.570 *	0.793 **	−0.895 **	−0.845 **	1

** correlation is significant at 0.01 level, * correlation is significant at 0.05 level, WAC—water absorption capacity, OAC—oil absorption capacity, SP—swelling power, EA—emulsion activity.

**Table 4:** Optimal sorghum flour compared to control sample.

Property	Optimal M		Control M
	Predicted	Experimental	
Protein (%)	8.74 ± 0.28 ^a^	9.66 ± 0.06 ^ax^	9.44 ± 0.18 ^x^
Fat (%)	4.16 ± 0.36 ^a^	4.26 ± 0.02 ^ax^	3.10 ± 0.03 ^y^
Ash (%)	1.96 ± 0.12 ^a^	2.00 ± 0.01 ^ax^	1.75 ± 0.03 ^y^
Moisture (%)	8.97 ± 0.08 ^a^	9.19 ± 0.01 ^ay^	11.80 ± 0.03 ^x^
Carbohydrates (%)	67.84 ± 0.61 ^a^	66.14 ± 0.10 ^ax^	66.00 ± 0.39 ^x^
Fiber (%)	8.33 ± 0.72 ^a^	9.02 ± 0.07 ^bx^	7.69 ± 0.39 ^y^
WAC (%)	203.02 ± 3.63 ^a^	217.00 ± 1.41 ^ax^	209.55 ± 0.78 ^y^
OAC (%)	171.09 ± 2.37 ^a^	179.63 ± 0.73 ^ax^	171.66 ± 0.60 ^y^
SP (g/g)	3.71 ± 0.06 ^a^	3.98 ± 0.01 ^ax^	3.63 ± 0.00 ^y^
EA (%)	41.28 ± 1.86 ^a^	42.27± 0.05 ^ax^	44.00 ± 1.41 ^x^

(a–b) different letters within same row for each sample indicate significant statistical differences between predicted and experimental values (*p* < 0.05); different letters (x–y) in same row indicate significant differences between optimal and control samples (*p* < 0.05). WAC—water absorption capacity, OAC—oil absorption capacity, SP—swelling power, EA—emulsion activity.

**Table 5:** Color parameters of control and optimal samples.

Bread Sample	Color Parameters					
	L*	a*	b*	C*	*BI*	ΔE
Control M	65.54 ± 0.55 ^a^	4.00 ± 0.03 ^a^	9.07 ± 0.10 ^a^	9.91 ± 0.10 ^a^	26.35 ± 0.04 ^a^	-
Optimal M	63.89 ± 0.08 ^b^	3.81 ± 0.13 ^b^	8.64 ± 0.12 ^b^	9.44 ± 0.06 ^b^	25.85 ± 0.11 ^b^	1.72

L*—lightness, a*—redness, b*—yellowness, C*—Chroma, *BI*—brown index, ΔE—total color difference; different letters (a–b) in same column indicate significant differences between optimal and control samples (*p* < 0.05).

**Table 6:** Thermal characteristics of optimal and control samples.

Sample	Ti (°C)	Tg (°C)	Tf (°C)	ΔT_r_ (°C)	ΔH (J/g)
Control M	69.03 ± 0.13 ^b^	73.35 ± 0.01 ^b^	79.58 ± 0.47 ^b^	10.55 ± 0.60 ^b^	1.13 ± 0.00 ^b^
Optimal M	69.82 ± 0.31 ^a^	73.40 ± 0.13 ^a^	82.07 ± 1.09 ^a^	12.25 ± 0.78 ^a^	2.15 ± 0.03 ^a^

T_i_—initial temperature, T_g_—gelatinization temperature, T_f_—final temperature, ΔT_r_—gelatinization range (ΔT_r_ =T_f_ − T_i_), ΔH—gelatinization enthalpy; different letters (a–b) in same coloumn indicate significant differences between optimal and control samples (*p* < 0.05).

**Table 7:** Starch digestibility of optimal and control samples.

Sample	RDS (g/100 g)	SDS (g/100 g)	TDS (g/100 g	RS (g/100 g)	TS (g/100 g)	SDRI
Control M	12.22 ± 0.02 ^a^	0.40 ± 0.01 ^b^	12.92 ± 0.02 ^b^	4.12 ± 0.00 ^b^	17.04 ± 0.02 ^b^	0.72 ± 0.00 ^a^
Optimal M	12.36 ± 0.07 ^a^	0.68 ± 0.00 ^a^	13.21 ± 0.04 ^a^	5.06 ± 0.03 ^a^	18.27 ± 0.08 ^a^	0.68 ± 0.00 ^b^

RDS—rapidly digested starch, SDS—slowly digested starch, TDS—total digestible starch, RS—resistant starch, TS—total starch, SDRI—starch digestion rate index; different letters (a–b) in same column indicate significant differences between optimal and control samples (*p* < 0.05).

**Table 8:** Total Phenolic Content and Antioxidant Activity of optimal and control samples.

Solvents	Samples	Total Phenolic Content (mg GAE/100 g)				Antioxidant Activity DPPH (%)			
		10 min	15 min	20 min	30 min	10 min	15 min	20 min	30 min
Water	Control M	10.32 ^fF^	10.63 ^fF^	11.18 ^eE^	11.80 ^dD^	86.88 ^cG^	88.20 ^cF^	88.44 ^cF^	89.97 ^cE^
	Optimal M	11.79 ^eD^	12.21 ^eC^	12.952 ^dB^	13.84 ^cA^	91.34 ^aD^	92.31 ^aC^	93.24 ^aB^	94.65 ^aA^
Metanol	Control M	15.90 ^bH^	16.54 ^bG^	17.06 ^bF^	17.59 ^bE^	89.59 ^bDE^	90.56 ^bC^	91.34 ^bB^	92.51 ^bA^
	Optimal M	22.22 ^aD^	22.74 ^aC^	23.16 ^aB^	24.85 ^aA^	89.31 ^bE^	90.17 ^bCD^	91.44 ^bB^	92.31 ^bA^
Ethanol	Control M	12.27 ^dG^	12.74 ^dF^	13.26 ^dE^	13.90 ^cE^	46.98 ^eE^	45.62 ^eF^	46.01 ^eF^	46.98 ^eE^
	Optimal M	15.47 ^cC^	15.47 ^cC^	16.63 ^cB^	17.47 ^bA^	77.33 ^dD^	78.69 ^dC^	80.05 ^dB^	81.42 ^dA^

Different letters (a–f) in same column represent significant statistical differences between type solvent values (*p* < 0.05), while different letters (A–G) in same row represent significant differences between time extraction values (*p* < 0.05). Data are expressed on dry matter (d.m.).

## Data Availability

All data are contained within the article.

## Supplementary Materials

The following supporting information can be downloaded via the following link: https://www.mdpi.com/article/10.3390/foods12112248/s1, Figure S1: DSC thermograms obtained for optimal and control sorghum flours; Table S1: D-optimal design and experimentally determined values of targeted responses: WAC (R1), OAC (R2), SP (R3), EA (R4), Protein (R5), Fat (R6), Ash (R7), Moisture (R8), Carbohydrates (R9) and Fiber (R10).
