# Supplementary material for: Sorghum Flour Features Related to Dry Heat Treatment and Milling
Source: Foods. 2023 Jun 2;12(11):2248. doi: 10.3390/foods12112248 (PMC10252988; doi:10.3390/foods12112248)
Supplement: Supplementary file 1 [file foods-12-02248-s001.zip › foods-2399066-table S1.pdf]

Table S1. The D-optimal design and experimentally determined values of targeted responses: Protein (R1); Fat (R2); Ash (R3); Moisture (R4); Carbohydrates (R5), Fiber (R6), WAC (R7); OAC (R8); SP (R9) and EA (R10).

Run	A: Treatment (°C)	B: Particle size (μm)	R1: Protein (%)	R2: Fat (%)	R3: Ash (%)	R4: Moisture (%)	R5: Carbohydrates (%)	R6: Fiber (%)	R7: WAC (%)	R8: OAC (%)	R9: SP (g/g)	R10: EA (%)
1	121.00	200.00	8.51	4.04	1.91	8.94	68.58	8.02	191.10	170.99	3.68	43.50
2	121.00	300.00	11.48	1.16	0.66	8.80	67.14	10.75	226.80	160.00	3.37	55.50
3	121.00	300.00	11.46	1.12	0.65	8.78	66.16	9.80	226.23	159.52	3.37	54.78
4	121.00	250.00	9.29	3.40	1.52	8.75	68.65	8.38	210.08	168.16	3.60	46.50
5	121.00	250.00	9.26	3.37	1.51	8.74	68.26	7.98	209.12	167.45	3.59	45.78
6	140.00	250.00	9.21	3.40	1.64	9.07	69.03	7.64	210.08	167.27	3.42	44.50
7	121.00	300.00	11.51	1.20	0.67	8.82	68.12	11.70	227.37	160.48	3.37	56.22
8	140.00	200.00	8.93	4.13	1.97	8.96	67.05	8.95	210.08	170.09	3.79	40.50
9	121.00	250.00	9.32	3.43	1.53	8.76	69.04	8.78	211.04	168.87	3.61	47.22
10	121.00	200.00	8.48	3.98	1.88	8.93	68.48	8.01	190.11	170.38	3.67	42.78
11	140.00	250.00	9.16	3.37	1.63	9.06	68.74	7.38	209.51	166.40	3.42	43.78
12	140.00	200.00	8.90	4.10	1.96	8.95	66.53	8.44	208.67	169.15	3.79	39.78
13	121.00	200.00	8.54	4.10	1.94	8.95	68.68	8.03	192.09	171.60	3.69	44.22
14	140.00	300.00	9.93	1.62	0.93	8.82	70.58	8.11	224.76	153.65	3.32	55.00
15	140.00	200.00	8.96	4.16	1.98	8.97	67.57	9.46	211.49	171.03	3.79	41.22
16	140.00	250.00	9.26	3.43	1.65	9.08	69.32	7.90	210.65	168.14	3.42	45.22
17	140.00	300.00	9.88	1.59	0.92	8.80	69.91	7.43	219.05	152.99	3.32	53.59
18	140.00	300.00	9.98	1.65	0.94	8.84	71.25	8.79	230.47	154.31	3.32	56.41

A—treatment temperature factor, B—particle size factor; WAC— water absorption capacity, OAC—oil-absorption capacity, SP—swelling power, EA—emulsion activity.
