# Supplementary figures and images for: Sorghum Flour Features Related to Dry Heat Treatment and Milling
Source: Foods. 2023 Jun 2;12(11):2248. doi: 10.3390/foods12112248 (PMC10252988; doi:10.3390/foods12112248)

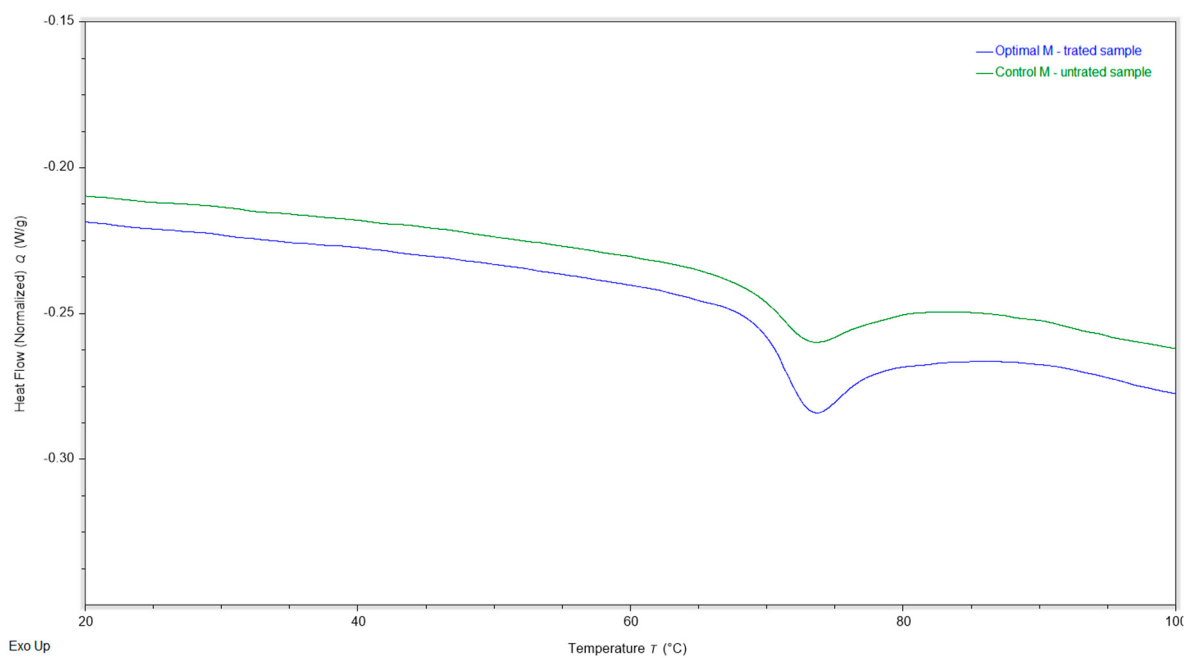

**Figure S1.** DSC thermograms obtained for optimal and control sorghum flours

Supplement: Supplementary file 1 [file foods-12-02248-s001.zip › foods-2399066-figure S1.pdf]
